# Improvement of Membrane Performances to Enhance the Yield of Vanillin in a Pervaporation Reactor

**DOI:** 10.3390/membranes4010096

**Published:** 2014-02-28

**Authors:** Giovanni Camera-Roda, Antonio Cardillo, Vittorio Loddo, Leonardo Palmisano, Francesco Parrino

**Affiliations:** 1 Dipartimento di Ingegneria civile, chimica, ambientale e dei materiali (DICAM), University of Bologna, via Terracini 28, Bologna 40131, Italy; E-Mail: antonio.cardillo2@unibo.it; 2 “Schiavello-Grillone” Photocatalysis group, Dipartimento di Energia, Ingegneria dell’Informazione e Modelli Matematici (DEIM), University of Palermo, viale delle Scienze, Palermo 90128, Italy; E-Mails: vittorio.loddo@unipa.it (V.L.); leonardo.palmisano@unipa.it (L.P.); francesco.parrino@unipa.it (F.P.)

**Keywords:** pervaporation reactors, vanillin, photocatalysis, mass transfer, process intensification

## Abstract

In membrane reactors, the interaction of reaction and membrane separation can be exploited to achieve a “process intensification”, a key objective of sustainable development. In the present work, the properties that the membrane must have to obtain this result in a pervaporation reactor are analyzed and discussed. Then, the methods to enhance these properties are investigated for the photocatalytic synthesis of vanillin, which represents a case where the recovery from the reactor of vanillin by means of pervaporation while it is produced allows a substantial improvement of the yield, since its further oxidation is thus prevented. To this end, the phenomena that control the permeation of both vanillin and the reactant (ferulic acid) are analyzed, since they ultimately affect the performances of the membrane reactor. The results show that diffusion of the aromatic compounds takes place in the presence of low concentration gradients, so that the process is controlled by other phenomena, in particular by the equilibrium with the vapor at the membrane-permeate interface. On this basis, it is demonstrated that the performances are enhanced by increasing the membrane thickness and/or the temperature, whereas the pH begins to limit the process only at values higher than 6.5.

## 1. Introduction

The techniques of production and/or separation of vanillin have been studied since its first utilization and production by the Totonac Indians [1]. The reasons of the importance of vanillin lie in the very special flavor of this aromatic aldehyde, which is appreciated on its own or in combination with other flavors. Furthermore, vanillin is also a powerful antioxidant with beneficial health effects. As a consequence, vanillin is widely utilized in several industries (food, cosmetic, nutraceutical, fine chemicals and pharmaceutical) for a world consumption of about 12,000 t/y [1,2,3,4,5,6].

At first, the investigation concerned only the curing methods of vanilla pods to enhance the formation of vanillin and the extraction procedures, but in the nineteenth century, the first isolation of vanillin from vanilla extract and the first chemical synthesis took place. It was the beginning of the industrial production of synthetic vanillin, whose share of the market has exceptionally risen since then. Currently, the natural production of vanillin is able to cover only a very small percentage (less than 1%) of the world demand, and the price of the vanillin produced from the pods of the tropical orchid (*Vanilla planifolia* or *Vanilla tahitensis*) is so high (about one hundred times the price of synthetic vanillin), that it is mainly utilized when there is the need to valorize the final product. On the other hand, the increasing consideration of consumers towards “green” and natural products [7,8,9,10] accelerated the research on alternative production methods. Among the possible solutions, the “natural” biosynthesis of vanillin [11,12,13,14,15,16,17,18] has been considered, and recently, also, a “green” photocatalytic synthesis has been proposed [19]. In these processes, the continuous recovery of vanillin directly from the reactor during its production, employing an “integrated” reaction-separation process, can significantly enhance the yield, so that a “process intensification” [20] is obtained. In fact, the removal of the aldehyde directly from the fermentation broth or from the reacting solution avoids any further degradation of the product in the reactor and any inhibition effect of vanillin on the reaction. To this aim, new separation processes must be considered, different from the ones (extraction by organic solvents, vacuum distillation and multi-stage recrystallization) that are traditionally utilized to extract vanillin from the vanilla pods or to purify the chemically synthesized or the natural product [6,21]. In fact, the operative conditions of the traditional methods of separation are not compatible with the mild conditions that must be adopted in the biochemical or in the photocatalytic processes. Under this aspect, membrane separation processes appear to be more appropriate, since they can operate at mild conditions. However, the membrane performances must be adequate to the special separation task, which is required in these “membrane reactors” for the production of vanillin. In particular, the membrane should be characterized by: (i) a sufficiently high flux, even at the relatively low vanillin concentrations that should be maintained in the reacting solution; (ii) satisfactory selectivity with respect to vanillin; and (iii) good rejection of the reactants. These qualities are often conflicting, and it is likely that very few systems can satisfy them. In fact, it is generally found that the permeation flux increases while the selectivity decreases. Furthermore, the precursors (substrates) that are utilized to produce vanillin are chemically similar to the product, and therefore, it is expected that also most of the chemical and physical properties, which generally determine the separation, are similar, so that there are few chances to find at least one property that is sufficiently different to be exploited for the membrane separation. In the case of vanillin, the attention of the researches has been mainly focused on pervaporation (PV), which looks to be a promising process to meet the previous requirements. Böddeker and coworkers [22,23,24] demonstrated that membranes in polyether-block-amide (PEBA) are very suitable for pervaporating low volatility aromatics [22] and, in particular, vanillin [24]. Brazinha *et al*. [25] analyzed the pervaporation of vanillin and ferulic acid with polyoctylmethylsiloxane (POMS) membranes, giving an explanation to the high separation factor of vanillin with respect to ferulic acid. Even if these pervaporation studies are finalized to the biosynthesis of vanillin, the adopted concentrations of vanillin are higher than those that should be reached in a membrane reactor to obtain the aforementioned advantages. Camera-Roda *et al*. [26] demonstrated that the yield of the production of aromatic aldehydes and, specifically, of vanillin [27] can be enhanced by the coupling of pervaporation with the photocatalytic reaction in the AROMA (Advanced Recovery and Oxidation Method for Aldehydes) process [28].

The aim of the present work is twofold: (i) to quantify by process simulation the effect of the enrichment factors and of the permeate flux on the yield in a PVR (pervaporation reactor), where vanillin is produced from ferulic acid; and (ii) to investigate the methods to enhance these pervaporation performances (enrichment factors and permeate flux) on the basis of the results obtained by process simulation. A pervaporation photocatalytic reactor [29,30] will be considered, since most of the drawn conclusions can be extended also to other pervaporation reactors, such as pervaporation bioreactors.

## 2. Materials and Methods

### 2.1. Experimental

Vanillin and trans-ferulic acid were purchased from Sigma-Aldrich (Saint Louis, MO, USA) with a purity >99%.

Commercial TiO_2_ Merck (100% anatase, Darmstadt, Germany) was used as the photocatalyst in a slurry suspension for the photocatalytic synthesis of vanillin using ferulic acid as the substrate.

The reacting solution was prepared by dissolving ferulic acid in deionized water at 60 °C and stirring for 30 min; then, Merck TiO_2_ powders were suspended in this solution and ultrasonicated for 15 min just before the experiments with the slurry photocatalytic reactor.

**Figure 1:**
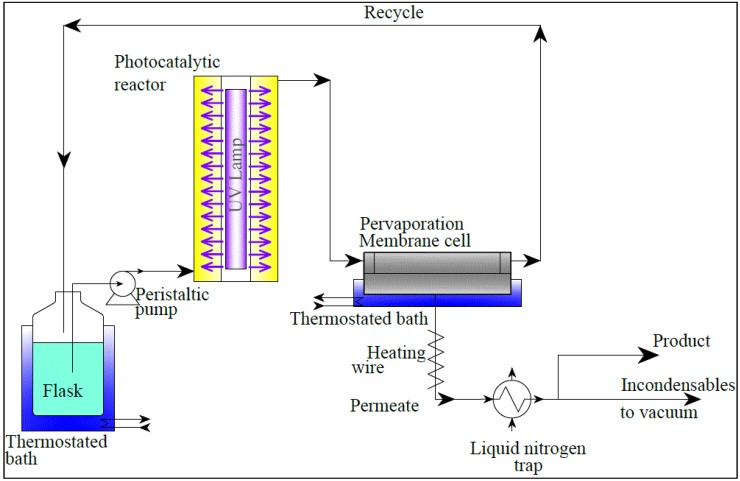
Schematic representation of the experimental apparatus.

Figure 1 illustrates the scheme of the apparatus. The fluid passes through an annular photocatalytic reactor and a pervaporation cell, where the permeate is separated into a pervaporate and a retentate. This latter enters a flask before being recycled by a peristaltic pump to the reactor. The flask and the pervaporation cell are thermostated. The pervaporation cell is immersed in a thermostated bath, and the tube connecting the pervaporation module to the condensation system is electrically heated to prevent condensation. The permeated vapors are finally condensed in a liquid nitrogen trap. The pressure of the permeate is kept at approximately 2–3 mbar with the aid of a vacuum pump.

Pervaporation (without photocatalysis) experiments were carried out with the same apparatus, simply by-passing the photocatalytic reactor from the recycle loop.

The tubes are in polytetrafluoroethylene and in Masterflex Chem-Durance^®^ (for the peristaltic pump, Vernon Hills, IL, USA) to avoid the adsorption of the organic compounds.

On the axis of the reactor, a linear fluorescent black light lamp (Philips 8W TL/08, Eindhoven, the Netherlands) emits UVA radiation from 350 to 400 nm. The glass walls in Pyrex minimize the absorption of the UVA radiation.

In the pervaporation cell, the flow between two parallel disks spaced 2 mm apart is radial from the center, with a flow rate of 70 L/h and an average velocity tangential to the membrane of 17.3 cm/s. The diameter of the part of the membrane exposed to the liquid is 7.45 cm for a permeating surface area of 43.6 cm^2^.

Additional details of the experimental apparatus and the analytical methods can be found in [27].

The preparation of the utilized membranes starts by dissolving overnight at 65 °C PEBAX^®^ 2533 (polyether block amide by Arkema) in *n*-butyl alcohol and isopropyl alcohol (PEBAX 8.5 wt %, *n*-butyl alcohol 23.0 wt %, isopropyl alcohol 68.5 wt %).

Then, a known volume of PEBAX solution is poured onto a Petri dish. The dish is put in a slightly ventilated oven, and it is horizontally levelled. In the oven, the solvents evaporate for one day at 70 °C. The detachment of the membrane from the Petri dish is facilitated by immersion of the dish with the membrane in deionized water for 4 h. The membrane is cut to obtain a circular membrane of a diameter of 9.5 cm. The membrane is then inspected under an optical microscope at five times magnification to detect possible defects (holes or thinner regions). Its thickness is measured in about twelve evenly distributed points by a Mitutoyo Digimatic 323–250 μm. Membranes with one defect or with an uneven thickness, that is, with just one point with a thickness differing more than ±15% with respect to the mean value, are discarded.

The final thickness of the dry membrane can be tuned by changing the quantity of the PEBAX solution poured onto the dish. For instance, 13 mL of the solution has been used to obtain a 60 µm-thick membrane on a Petri dish with a 14.2 cm diameter.

### 2.2. Process Simulation

In order to assess the importance of the separation properties in a PVR, the effects of the flux and of the enrichment factors on the yield of the integrated continuous process have been investigated by process simulation, a well-established method, which is widely used to this aim.

**Figure 2:**
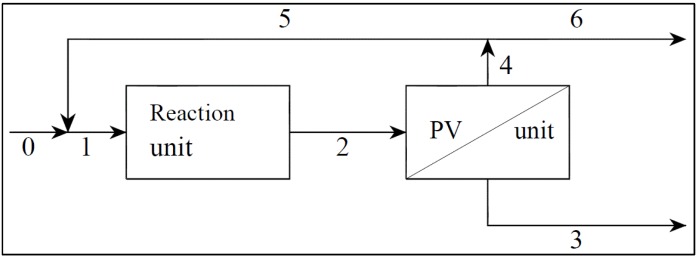
Scheme of the integrated photocatalysis-pervaporation (PV) continuous process.

The layout adopted in the present process simulation is shown in Figure 2. The integration of photocatalysis with membrane separation is achieved using separate units, thanks to the recycle loop, which gives the necessary back mixing from the membrane separation unit to the reactor, so that photocatalysis and pervaporation actually operate on the same solution [30]. The fluid passes through the reaction unit and the PV unit, which separates a permeate (Stream 3) from a retentate (Stream 4). The retentate is divided by a splitter into two streams, one of which is recycled to the reactor (Stream 5), while the other one is purged (Stream 6); then, a mixer merges Streams 0 and 5 into Stream 1. The recycle ratio, *R*, is here defined as the flow rate of Stream 5 to the flow rate of Stream 2. An important advantage deriving from the adoption of separate units is a larger degree of freedom [31], which ultimately provides the possibility of optimizing a larger number of parameters and, consequently, obtaining a more important process intensification.

In the complex chemically reacting system, only the reactions that affect the yield of vanillin will be considered for the present investigation.

The simplified set of reactions is:

S→P Reaction 1, which produces the desired product P (vanillin) from the substrate, S (ferulic acid), by photocatalytic partial oxidation with a reaction rate *R*_1_ = *K*_1_ *C_S_*;
S→B Reaction 2, which, in parallel, produces the unwanted Product B with a reaction rate *R*_2_ = *K*_2_ *C_S_*;
P→C Reaction 3, which degrades vanillin by a further oxidation with a reaction rate *R*_3_ = *K*_3_ *C_P_*.

Because of the relatively low concentration of the reactants, a first order can be assumed for all the photocatalytic reactions in the system [32]. In the reacting solution, the parallel reactions, which involve S as the reactant, are more than one, but since the study is limited to vanillin production, the parallel reactions different from Reaction 1 can be lumped into only one pseudo Reaction 2, with an overall reaction rate given by the sums of the rates of the parallel reactions. Furthermore, the other consecutive reactions, which do not involve vanillin, can be ignored.

For each unit in the scheme (mixer, reactor, pervaporation unit and splitter), the steady-state material balances together with the relevant relationships, which describe physical and chemical phenomena, have been considered and solved. The resulting system of non-linear algebraic equations can be found in [33]. It is solved iteratively with the successive substitutions method or, in some cases, with the Newton-Raphson method. The yield in vanillin can then be computed as yield:

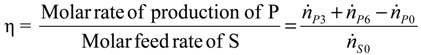

where *ṅ_ij_* is the molar rate of the chemical species *i* (*i =* S, P) in stream *j*.

The dimensionless parameters, which are obtained by the dimensional analysis of the set of the governing equations, are listed below.

The following is a list of the dimensionless parameters:

- the ratios, *R*_2_ = *k*_2_/*k*_1_ and *R*_3_ = *k*_3_/*k*_1_, between kinetic constants;
- the enrichment factors of the membrane, β_S_ = *C_S_*_,permeate_/*C_S_* and β_P_ = *C_P_*_,permeate_/*C_P_* , where *C_i_* is the concentration of the permeating compound upstream of the membrane and *C_i_*_,permeate_ is the concentration in the condensed permeate;
- the recycle ratio, 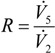 (0 ≤ *R* < 1), of the flow rate of Stream 5 to the flow rate of Stream 2;
- the Damköhler number, 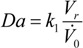, where *V_r_* is the volume of the reactor and 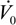 is the volumetric flow rate of stream 0;
- the Péclet number, 
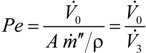
, where *A* is the membrane area, ρ is the density of the condensed permeate and 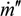 is the total mass flux of the permeate through the membrane. The present definition of the Péclet number is consistent with the one commonly adopted in membrane reactors (see, e.g., [34,35]), with *Pe* representing the ratio of the convective transport to the permeation rate through the membrane. The highest allowable value of the reciprocal of *Pe*, 1/*Pe*, is 1, and it is reached when the membrane area is so high as to attain a flow rate of the permeate that equals the flow rate of the fresh feed to the system (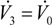 and 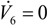), while the lowest value is 0, which represents the case of a photocatalytic reactor without pervaporation (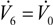 and 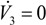).

It is apparent that, for a given reacting system with the given kinetics and properties of the membrane, the Damköhler number and the reciprocal of the Péclet number, 1/*Pe*, are proportional to the volume of the reactor and to the membrane area, respectively. Furthermore, from the present definition of the Péclet number, it follows that, if the permeate flux is enhanced, a lower membrane area is sufficient to achieve a certain value of 1/*Pe*, or alternatively, a higher value of 1/*Pe* is obtained for a given membrane area.

Kinetic constants and membrane properties (flux and enrichment factors) have been obtained experimentally by Camera-Roda *et al*. [27]. The resulting values of the relevant parameters are (see [27]): *R*_2_ = 6.8, *R*_3_ = 8.7, β_S_ = 0.005 and β_P_ = 4.2. Even if these values can be varied to some extent by changing the operating conditions or the preparative conditions of the membrane, they will be used as reference values in the process simulation, unless otherwise indicated.

Given that *R*_3_, the ratio of the characteristic rate of degradation of vanillin (Reaction 3) to the one of production (Reaction 1), is much higher than 1; then, it is highly advisable to recover the desired product directly from the reacting solution [36]. The enrichment factor of S is much less than 1, indicating that its rejection is close to 1, and the losses of S in the permeate stream are small.

In the photocatalytic reacting system, vanillin is an intermediate valuable compound of reactions in series, so that its concentration reaches a maximum at an optimal residence time [36], which corresponds to an optimal value of *Da*.

An increase of the recycle ratio, *R*, increases the effectiveness of the integration of reaction with pervaporation, with positive effects on the yield [30,33], but increases also the degree of backmixing, with negative effects on the yield of an intermediate product [36], such as vanillin. Consequently, an optimal value must be expected also for *R*. This means that the yield is maximized for an optimal couple of values of both *Da* and *R*.

## 3. Results and Discussion

### 3.1. Results Obtained by the Process Simulation

The investigation is carried out by taking into consideration the maximum achievable yield, η_max_, which is obtained in the different cases at relevant optimal values of *R* and *Da*.

**Figure 3:**
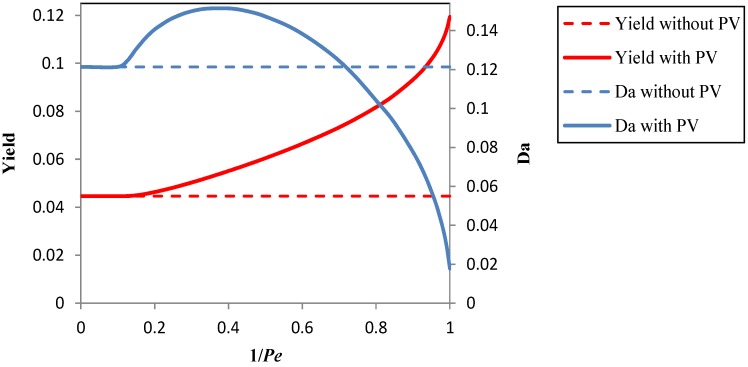
The maximum yield at the optimal Damköhler number *vs*. the reciprocal of the Péclet number in the integrated photocatalysis-pervaporation process and without pervaporation. β_S_ = 0.005, β_P_ = 4.2. *Pe*, Péclet number.

Figure 3 shows the maximum yield and the associated optimal Damköhler number *versus* the reciprocal of the Péclet number, 1/*Pe*, with and without the coupling with the pervaporation unit. Thanks to the coupling, the process is “intensified”, and η_max_ (obtained at 1/*Pe* = 1) becomes equal to 0.119, which represents a 168% increase in comparison with the maximum value that can be obtained without pervaporation (η_max_ = 0.0446). It is apparent that: (i) the yield increases with 1/*Pe*, that is, with the available membrane area; and (ii) a minimum value of the membrane area must be exceeded to get an increase of the yield.

In the hypothesis that it might be possible to increase the separation capabilities of the pervaporation process, it is interesting to preliminarily investigate how the membrane properties could affect the performances of the integrated process.

**Figure 4:**
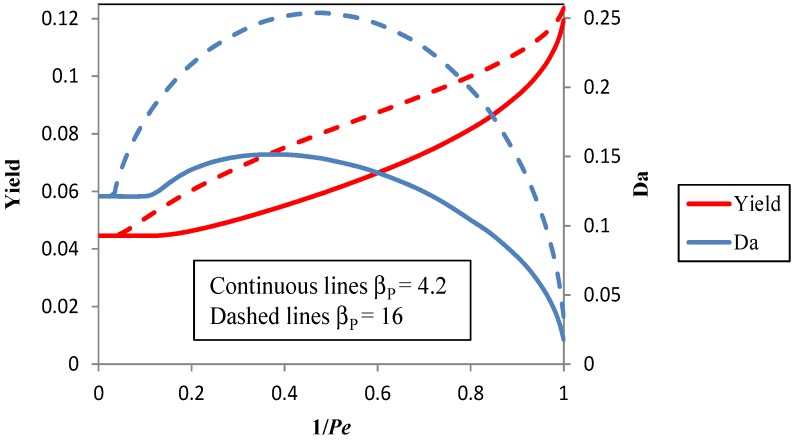
The maximum yield at the optimal Damköhler number *vs*. the reciprocal of the Péclet number in the integrated photocatalysis-pervaporation process for β_P_ = 4.2 and β_P_ = 16, β_S_ = 0.005.

In Figure 4, the yield and the optimal value of *Da versus* the reciprocal of *Pe* are presented for the reference value (β_P_ = 4.2) and for a membrane with an enrichment factor of vanillin β_P_ = 16. At *Pe* = 1, only a slight enhancement of the yield is obtained; in fact, the maximum yield passes from 0.1194 for β_P_ = 4.2 to 0.1235 for β_P_ = 16 with a small 3.5% increase, but it is evident that the enhancement is more important at intermediate values of 1/*Pe*. From another point of view, an improvement of the enrichment factor of vanillin provides a significant savings in membrane area to get a given yield. For instance, if the target is to get an 8% yield, when β_P_ = 4.2, the membrane area is 64% larger than the one necessary when β_P_ = 16, since concurrently, 1/*Pe* decreases from 0.781 to 0.476.

**Figure 5:**
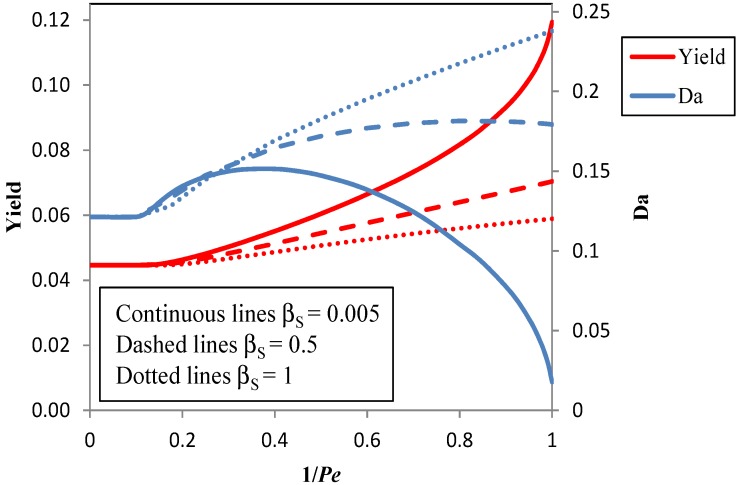
The maximum yield at the optimal Damköhler number *vs*. the reciprocal of the Péclet number in the integrated photocatalysis-pervaporation process for β_S_ = 0.005, β_S_ = 0.5 and β_S_ = 1, β_P_ = 4.2.

Figure 5 shows the effect of a change of the enrichment factor of the reactant (ferulic acid) from 0.005 to one on the achievable yield.

The rejection, *R_S_*, of S by the membrane is directly related to the enrichment factor, since *R_S_* = 1 − β_S_ [37]. A high rejection of the reactant is advantageous, since it reduces the losses of the reactant in the permeate. The results in Figure 5 show that the achievable yield is severely affected by a low rejection of S, in particular, at high values of 1/*Pe*, that is, at large values of the membrane area. Furthermore, when the rejection is low, the yield is maximized at very large values of the Damköhler number, so that large volumes of the reactor are needed.

In summary, process simulation shows why it is important to choose or to develop membranes that selectively permeate the product (β_P_ >> 1), but also retain the reactant (β_S_ << 1). This requirement implies that it is not sufficient that the membrane be able to selectively separate P from S. In parallel, a relatively high vanillin permeate flux should be pursued, since it allows one to operate at higher values of 1/*Pe*, even with a limited membrane area. In the following section, some methods to improve these membrane properties are analyzed.

### 3.2. Improvement of the Pervaporation Performances of PEBA Membranes

Primarily, it is necessary to determine the factors that affect the membrane properties, whose importance has been established in the previous section. Valuable indications can be obtained from previous studies. Indeed, the pervaporation of aqueous solutions containing vanillin, its precursor and possibly other products of the reaction have already been studied through PEBA [24,27] and POMS [25] membranes. Some interesting behaviors were observed, such as a relative independence of the vanillin flux on the membrane thickness, *l*, whereas the water flux significantly decreases with *l*, and an exponential increase of the vanillin flux with temperature. Furthermore, Brazinha *et al*. [25] suggested that pH plays an important role in limiting the permeation of the reactant and of vanillic acid, which is a by-product of the reacting system. 

On this basis, it is expected that the membrane thickness, the temperature and the pH could influence the fluxes and the enrichment factors, and for this reason, their effects are here investigated.

**Figure 6:**
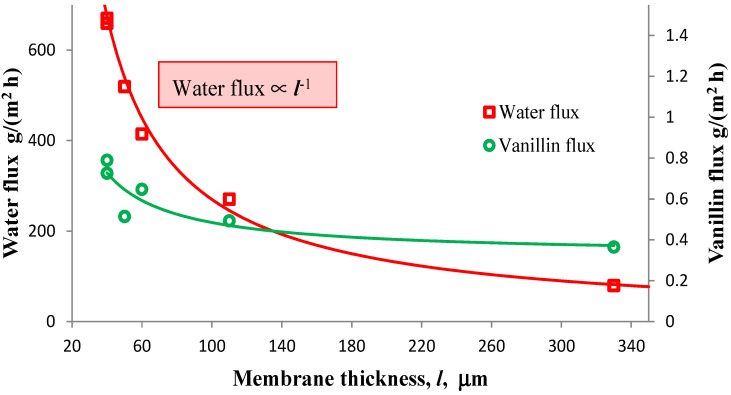
Water and vanillin fluxes *vs*. the thickness, *l*, of the membrane. *T* = 60 °C, permeate pressure = 2 mbar, feed concentration of vanillin = 420 ppm.

Figure 6 shows the dependence of the fluxes of vanillin and water on the membrane thickness, *l*.

The percentage decrease of the vanillin flux is much less than the one of the water flux, in particular when the membrane thickness is increased from 50 to 330 μm. The water flux follows the typical proportionality with *l*^−1^, which is predicted by the solution-diffusion model [38]. As a consequence, it is possible to increase the enrichment factor of vanillin, without significantly affecting its flux, simply by increasing the membrane thickness. In fact, the enrichment factor passes from approximately 3.1 when *l* = 60 μm to about 10.8 when *l* = 330 μm. It must be pointed out that the same behavior was observed also by Böddeker [24]. A possible explanation is that the resistance to the vanillin transport through the membrane is so low that it limits the permeation only slightly, even in the presence of relatively thick membranes. If this hypothesis is true, the driving force, which is given by the difference of the partial pressure of vanillin between the feed and the permeate, would be small. This occurrence can be verified from experimental data.

In an experiment carried out permeating a 1000 ppm (*x*_van_ = 1.18 × 10^−4^) aqueous solution of vanillin with a 60 μm-thick membrane at 60 °C and at a permeate pressure of 2 mbar, the measured vanillin mole fraction in the vapor permeate was *y*_van_ = 3 × 10^−4^. Therefore, the partial pressure of vanillin at the permeate side is *P*_van,permeate_ = 2 × 3 × 10^−4^ = 6 × 10^−4^ mbar.

The partial pressure of vanillin at the feed side can be evaluated as 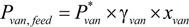, where 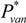 is the vapor pressure of vanillin and γ_van_ is the activity coefficient of vanillin.

Values of the vapor pressure of vanillin at temperatures ranging from 0 °C to 20 °C have been obtained from the composition at 2 mbar of the vapor permeate containing water and vanillin in equilibrium, with pure vanillin crystals deposited at these temperatures. Then, the value of the vapor pressure at 60 °C has been estimated by extrapolating these data by the Antoine equation, which was shown to reproduce the experimental results very accurately. The calculated vapor pressure is 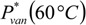 = 0.1105 mbar. The activity coefficient, computed with the UNIFAC group contribution method, is γ*_van_* = 47.18 at *T* = 60 °C and *x*_van_ = 1.18 × 10^−4^.

Therefore, the partial pressure at the feed side is *P*_van,feed_ = 0.1105 × 47.18 × 1.18 × 10^−4^ = 6.17 × 10^−4^ mbar and the driving force is *P*_van,feed_ − *P*_van,permeate_ = 0.17 × 10^−4^ mbar, which is indeed a very low value, even taking into account the uncertainty of the evaluation of the activity coefficient and of the vapor pressure. This outcome indicates that pervaporation of vanillin takes place in the presence of a small gradient of concentration of vanillin, and this is in accordance with the results presented by Böddeker *et al*. [22] for the “pervaporation of low volatility aromatics from water” with PEBA membranes. Furthermore, the diffusion coefficient of vanillin in these PEBA membranes has been estimated as 9 × 10^−11^ m^2^/s in a dialysis experiment similar to the one described in [39]. This value is at least one order of magnitude higher than the diffusivity of phenol in PEBA estimated by Böddeker *et al*. [23], and for the experimentally observed fluxes in pervaporation, it is absolutely compatible with an almost flat vanillin concentration profile inside the membrane. The high diffusivity of vanillin confirms that mass transfer in the membrane has only a minor role in the control of vanillin permeation and that it is possible to increase the membrane thickness in order to enhance the enrichment factor without severely affecting the vanillin flux. Besides, the concurrent decrease of the water flux determines a savings of the energy required to evaporate water at the permeate side and, possibly, an easier purification of vanillin from the more enriched permeate.

**Figure 7:**
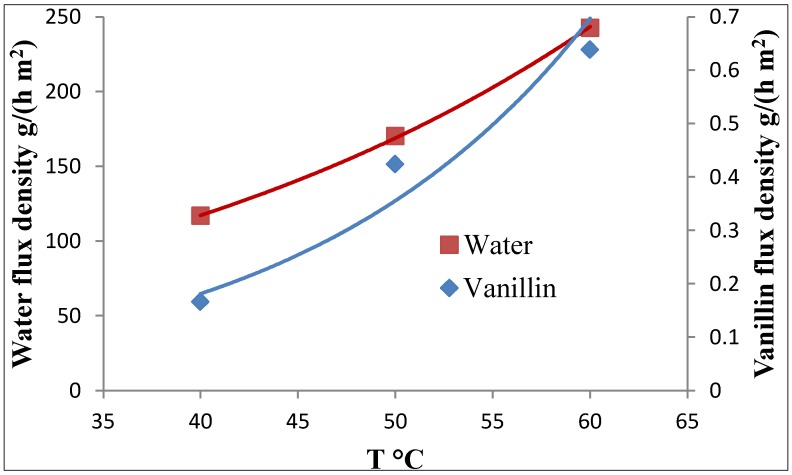
Water and vanillin fluxes *vs*. the pervaporation temperature. Feed concentration of vanillin = 420 ppm, permeate pressure = 2 mbar, *l* = 70 μm.

The effects of the temperature on the vanillin and water fluxes are shown in Figure 7.

The activation energy for the vanillin flux (*E*_van_ = 58.6 kJ/mol) is higher than the one of water (*E*_water_ = 31.7 kJ/mol). Hence, both the vanillin flux and the enrichment factor of vanillin increase with the temperature. By the way, the variation of the vanillin flux with the temperature is very similar to the variation with the temperature of the vanillin vapor pressure, thus suggesting that the equilibrium of vanillin at the interface between the membrane phase and the permeate vapor is limiting the permeation of vanillin. It is worth noting that photocatalytic pervaporation reactors can tolerate relatively high temperatures better than pervaporation bioreactors [30,40].

As previously mentioned, the possible importance of the pH in the separation of vanillin from the reactant and the by-products by pervaporation was hypothesized by Brazinha *et al*. [25]. They observed that in POMS membranes at the usual pH (pH ≈ 7.2), which holds in the fermentation broths for the bioproduction of vanillin, the fluxes of the substrate (ferulic acid) and of vanillic acid (a by-product) are almost negligible, whereas the vanillin permeates to some extent. They suggested that the rejection could be due to the complete dissociation of ferulic acid and vanillic acid, whose pK_a_ is around 4.5. As charged species, they are not volatile; so, the evaporation is inhibited and, consequently, the permeation is very limited. On the contrary, at pH = 7.2, vanillin, thanks to the higher value of the pK_a_ (pK_a_ ≈ 7.78), is only partially dissociated, so it can permeate.

**Figure 8:**
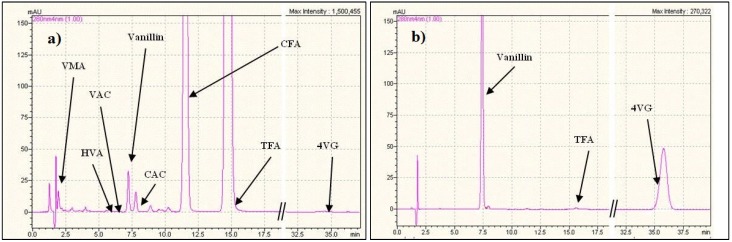
HPLC chromatograms: (**a**) pervaporation feed (a photocatalytic reacting solution after two hours of reaction); (**b**) permeate of the same solution. VMA, Vanillylmandelic acid; HVA, homovanillic acid; VAC, vanillic acid; CAC, caffeic acid; CFA, cis-ferulic acid; TFA, trans-ferulic acid; 4VG, 4-vynil guaiacol. The other peaks are unknown. *T* = 60 °C, permeate pressure = 2 mbar, *l* = 40 μm, pH = 5.5.

This hypothesis can be checked in the photocatalytic synthesis of vanillin from ferulic acid. Firstly, the permeation and the rejection of the membrane towards vanillin, ferulic acid and vanillic acid in PEBA membranes is assessed by examining the HPLC chromatograms of the feed and of the permeate, which are shown in Figure 8. It must be observed that in the reacting mixture, many other by-products are present, such as vanillylmandelic acid, homovanillic acid, caffeic acid and 4-vynil guaiacol.

Despite the very large number of peaks that are present in the feed, only a higher vanillin peak, an almost vanishing trans-ferulic acid (TFA) peak and a much higher 4-vynil guaiacol (4VG) peak appear in the permeate. This means that the membrane is able to retain with a very high rejection almost all the compounds, except vanillin and 4VG, which, on the contrary, are enriched in the permeate. For instance, vanillin concentration in the condensed permeate (15.8 ppm) is about 4.3 times higher than in the reacting solution (3.7 ppm).

**Figure 9:**
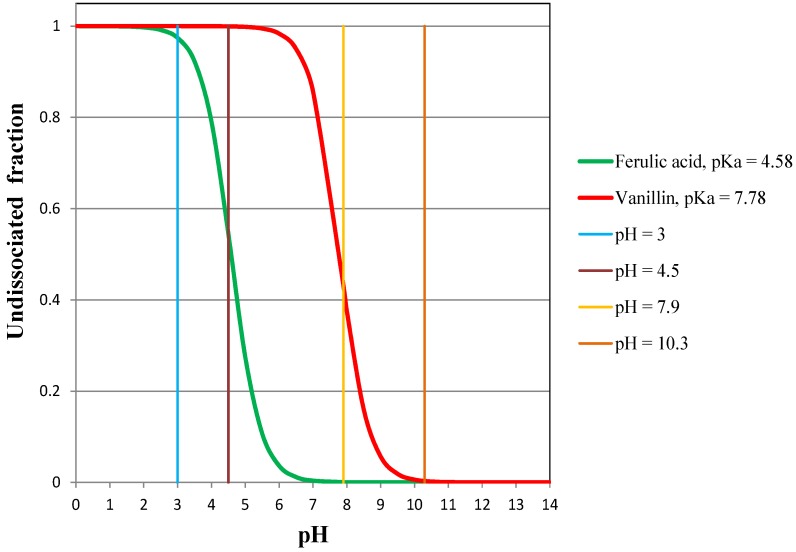
Undissociated fractions of vanillin and ferulic acid in aqueous solution *vs*. pH.

To verify the effect of the pH on the permeation and the rejection of vanillin and ferulic acid, pervaporation experiments of solutions containing vanillin and trans-ferulic acid were carried out at four different values of pH: pH = 3 with both vanillin and TFA totally undissociated; pH = 4.5 with vanillin totally undissociated and TFA partially dissociated; pH = 7.9 with vanillin partially dissociated and ferulic acid completely dissociated; and pH = 10.3 with both vanillin and TFA completely dissociated. The values of the undissociated fractions of vanillin and ferulic acid *versus* pH can be easily computed from their pKa values using the Henderson-Hasselbalch equation. The relevant curves thus obtained are shown in Figure 9.

The results of the pervaporation experiments at different pH values are summarized in Table 1.

**Table 1:** The flux of vanillin (Van) and trans-ferulic acid (TFA) at different values of pH. Temperature = 60 °C, permeate pressure = 2 mbar, δ = 60 μm.

pH	Feed concentrations	Flux vanillin	Flux TFA
	ppm	g/(h m^2^)	g/(h m^2^)
3	609 Van + 777 TFA	0.64	2.74 × 10^−3^
4.5	600 Van + 388 TFA	0.64	4.50 × 10^−4^
7.9	540 Van + 660 TFA	0.28	3.20 × 10^−4^
10.3	609 Van + 774 TFA	0.01	1.63 × 10^−4^

These experimental results show that:

- the flux of vanillin does not change as long as it is totally undissociated (pH = 3 and pH = 4.5);
- the flux of vanillin is reduced to a certain degree when it is partly dissociated (pH = 7.9);
- the flux of vanillin is drastically reduced when it is dissociated (pH = 10.3);
- the higher the pH (or the higher the dissociated fraction of TFA), the lower the permeate flux of TFA;
- when TFA is totally undissociated, its flux is highly enhanced (at pH = 3, the flux is one order of magnitude higher than at pH = 10.3), but it still remains absolutely negligible.

As expected, the dissociation affects the permeation, but as a matter of fact, it has a minor practical effect on the obtainable performances in the pervaporation reactor. In fact, the permeation of vanillin can be limited by the dissociation only when the dissociation is significant, in other words, at relatively high values of pH, and the permeation of the ferulic acid is very low, even when it is completely undissociated. With regard to the necessity of avoiding the dissociation of vanillin, it should be advisable not to exceed pH 6.5, to be sure that the dissociated fraction of vanillin is negligible. Indeed, the presence of a dissociated fraction of vanillin hinders, to some extent, the rate of permeation, since it reduces the driving force for the permeation of the undissociated fraction, albeit the dissociated form is continuously transformed into the undissociated form, due to the continuous recovery of this latter by pervaporation [25]. In this respect, the photocatalytic synthesis of vanillin is more suitable to work at low pH than the biotechnological processes.

Taking into consideration the previous observations, it is clear that other phenomena, different from the dissociation of the compounds in aqueous solution, should actually limit the permeation of ferulic acid. It is very likely that the volatility of the permeating species, even when undissociated, plays a fundamental role in the pervaporation of these “low volatility” compounds. Even if the volatility of vanillin is relatively low, however, it is much higher than the one of TFA. For instance, at 25 °C, the vapor pressure of vanillin (2.2 × 10^−3^ mbar) is nearly three orders of magnitude higher than the one of TFA (3.6 × 10^−6^ mbar), and the boiling point of vanillin (285 °C) is much lower than the one of TFA (372 °C). Most of the by-products have a volatility lower than the one of vanillin, as is confirmed by their boiling points, which are well beyond the boiling point of vanillin: 421 °C for vanillylmandelic acid, 368.7 °C for homovanillic acid, 353.4 °C for vanillic acid and 366 °C for caffeic acid. Therefore, these compounds can possibly diffuse through the membrane, but they cannot pass into the permeate vapors, since their evaporation is very limited by the equilibrium at the membrane-permeate interface. On the contrary, 4-vynil guaiacol is more volatile than vanillin, as its boiling point (224 °C) is lower than 285 °C and its pKa is high (pKa ≈ 10). Consequently, its concentration in the permeate is significant, even when only traces are present in the feed (see the chromatograms in Figure 8).

In order to eliminate the possible limitation caused by the evaporation at the membrane-permeate interface, some experiments with the same dense PEBA membranes have been carried out in a dialysis process, where, also, the permeate is a liquid aqueous solution, so that permeation can take place without the evaporation of the permeate. The preliminary results show that an almost tenfold increase of the vanillin flux is observed with respect to the one obtained in pervaporation under similar operating conditions, and the permeability of ferulic acid and of the other by-products becomes comparable to the permeability of vanillin. These latter outcomes confirm that: (i) all these aromatic compounds can easily diffuse through the membrane; (ii) the low volatility of the higher boiling point compounds is the cause of their high retention in pervaporation; and (iii) in pervaporation, the permeation of vanillin is somehow limited by its relatively low volatility.

## 4. Conclusions

Vanillin is an intermediate compound of consecutive reactions, whose production can be highly enhanced in a pervaporation reactor, where pervaporation recovers vanillin directly from the reacting solution, thus avoiding any further degradation. In order to accomplish this task, it has been demonstrated by process simulation that the main requisites are: (i) a high enrichment factor of vanillin, whose main effects are an enhancement of the yield and/or a reduction of the required membrane area; (ii) a high rejection of the reacting substrate (ferulic acid), whose main effects are an enhancement of the conversion and a reduction of the required volume of the reactor; and (iii) a relatively high flux of vanillin, whose main effects are a reduction of the required membrane area to get a given yield or an increase of the yield if the membrane area is kept constant. PEBA-dense membranes appear suitable to be used in pervaporation reactors, where vanillin is being produced. The study of the vanillin pervaporation with PEBA membranes shows that a viable method to enhance the enrichment factor of vanillin is to increase the membrane thickness, since the resistance to vanillin permeation remains low while the resistance to water permeation increases. An improvement of the enrichment factor can be obtained also by raising the temperature, with the additional positive effect of increasing the vanillin flux. Finally, the real reason for the very high rejection of the substrate (ferulic acid) and of most of the by-products is the low volatility of these compounds. The pH has a minor influence on the rejection of the substrate, which remains high also at low pH, when the substrate in solution is not dissociated. Conversely, the permeation of vanillin is affected by pH. In fact, permeation of vanillin is relatively high if vanillin is not dissociated (pH < 6.5), but it decreases at pH = 7.9 when vanillin is partially dissociated and becomes particularly low when vanillin is completely dissociated at pH = 10.3.

## References

[1] Havkin-Frenkel D., Belanger F.C. (2011). Handbook of Vanilla Science and Technology.

[2] Dignum M.J.W., Kerler J., Verpoorte R. (2000). Vanilla production: Technological, chemical and biosynthetic aspects. Food Rev. Int..

[3] Walton N.J., Mayer M.J., Narbad A. (2003). Vanillin. Phytochemistry.

[4] Esposito L., Formanek K., Kientz K.G., Mauger F., Maureaux V., Robert G., Truchet F. (1997). Vanillin. Kirk-Othmer Encyclopedia of Chemical Technology.

[5] Korthou H., Verpoorte R. (2007). Vanilla. Flavours and Fragrances.

[6] Ramachandra R.S., Ravishankar G.A. (2000). Vanilla flavour: production by conventional and biotechnological routes. J. Sci. Food Agric..

[7] Cheetham P.S.J. (1997). Combining the technical push and the business pull for natural flavours. Adv. Biochem. Eng. Biotechnol..

[8] Straughan R.D., Roberts J.A. (1999). Environmental segmentation alternatives: A look at green consumer behavior in the new millennium. J. Consum. Market..

[9] Laroche M., Bergeron J., Barbaro-Forleo G. (2001). Targeting consumers who are willing to pay more for environmentally friendly products. J. Consum. Market..

[10] Pickett-Baker J., Ozaki R. (2008). Pro-environmental products: Marketing influence on consumer purchase decision. J. Consum. Market..

[11] Serra S., Fuganti C., Brenna E. (2005). Biocatalytic preparation of natural flavours and fragrances. Trends Biotechnol..

[12] Longo M.A., Sanromán M.A. (2006). Production of food aroma compounds: Microbial and enzymatic methodologies. Food Technol. Biotechnol..

[13] Muheim A., Lerch K. (1999). Towards a high-yield bioconversion of ferulic acid to vanillin. Appl. Microbiol. Biotechnol..

[14] Gounaris Y. (2010). Biotechnology for the production of essential oils, flavours and volatile isolates. A review. Flavour Frag. J..

[15] Vandamme E.J., Soetaert W. (2002). Bioflavours and fragrances via fermentation and biocatalysis. J. Chem. Technol. Biotechnol..

[16] Kumar R., Singh S., Singh O.V. (2008). Bioconversion of lignocellulosic biomass: Biochemical and molecular perspectives. J. Ind. Microbiol. Biotechnol..

[17] Schwab W., Davidovich-Rikanati R., Lewinsohn E. (2008). Biosynthesis of plant derived flavor compounds. Plant J..

[18] Berger R.G. (2009). Biotechnology of flavours—The next generation. Biotechnol. Lett..

[19] Augugliaro V., Camera-Roda G., Loddo V., Palmisano G., Palmisano L., Parrino F., Puma M.A. (2012). Synthesis of vanillin in water by TiO_2_ photocatalysis. Appl. Catal. B Environ..

[20] Stankiewicz A., Moulijn J.N. (2000). Process intensification transforming chemical engineering. Chem. Eng. Progr..

[21] Converti A., Aliakbarian B., Domìnguez J.M., Bustoz Vàsquez G., Perego P. (2010). Microbial production of vanillin. Braz. J. Microbiol..

[22] Böddeker K.W., Bengston G., Bode E. (1990). Pervaporation of low volatility aromatics from water. J. Membr. Sci..

[23] Böddeker K.W., Bengston G., Pingel H., Dozel S. (1993). Pervaporation of high boilers using heated membranes. Desalination.

[24] Böddeker K.W., Gatfield I.L., Jähnig J., Schorm C. (1997). Pervaporation at the vapor pressure limit: Vanillin. J. Membr. Sci..

[25] Brazhina C., Barbosa B., Crespo G.J. (2011). Sustainable recovery of pure natural vanillin from fermentation media in a single pervaporation step. Green Chem..

[26] Camera-Roda G., Santarelli F., Augugliaro V., Loddo V., Palmisano G., Palmisano L., Yurdakal S. (2011). Photocatalytic process intensification by coupling with pervaporation. Catal. Today.

[27] Camera-Roda G., Augugliaro V., Cardillo A., Loddo V., Palmisano G., Palmisano L. (2013). A pervaporation photocatalytic reactor for the green synthesis of vanillin. Chem. Eng. J..

[28] Camera-Roda G., Augugliaro V., Loddo V., Palmisano G., Palmisano L. (2011). Production of Aldehydes by Oxidation in Aqueous Medium with Recovery of the Product by Means of Pervaporation. U.S. Patent.

[29] Sanchez Marcano J.G., Tsotsis T.T. (2002). Pervaporation Membrane Reactors. Catalytic Membranes and Membrane Reactors.

[30] Camera-Roda G., Augugliaro V., Loddo V., Palmisano L., Basile A. (2013). Pervaporation Membrane Reactors. Handbook of Membrane Reactors.

[31] Schembecker G., Tlatlik S. (2003). Process synthesis for reactive separations. Chem. Eng. Process..

[32] De Lasa H., Serrano B., Salaices M. (2005). Photocatalytic Reactor Engineering.

[33] Camera Roda G., Santarelli F. (2012). Design of a pervaporation photocatalytic reactor for process intensification. Chem. Eng. Technol..

[34] Battersby P.W., Teixeira P.W., Beltramini J., Duke M.C., Rudolph V., Diniz Da Costa J.C. (2006). An analysis of the Péclet and Damköhler numbers for dehydrogenation reactions using molecular sieve silica (MSS) membrane reactors. Catal. Today.

[35] Moon W.S., Park S.B. (2000). Design guide of a membrane for a membrane reactor in terms of permeability and selectivity. J. Membr. Sci..

[36] Levenspiel O. (1999). Chemical Reaction Engineering.

[37] Böddeker K.W. (2008). Liquid Separation with Membranes.

[38] Wijmans J.G., Baker R.W. (1995). The solution-diffusion model: A review. J. Membr. Sci..

[39] Groß A., Heintz A. (2000). Diffusion coefficients of aromatics in nonporous PEBA membranes. J. Membr. Sci..

[40] Vane L.M. (2005). A review of pervaporation for product recovery from biomass fermentation processes. J. Chem. Technol. Biotechnol..

